# Anxiety and Depression Affect Early Postoperative Pain Dimensions after Bariatric Surgery

**DOI:** 10.3390/jcm10010053

**Published:** 2020-12-25

**Authors:** Sofia Gravani, Maria Matiatou, Pantelis T. Nikolaidis, Evangelos Menenakos, Constantinos G. Zografos, George Zografos, Konstantinos Albanopoulos

**Affiliations:** 1 1st Propaedeutic Surgical Department, Medical School, National and Kapodistrian University of Athens, Hippocratio General Hospital of Athens, 11527 Athens, Greece; m.matiatou@gmail.com (M.M.); surg-clinic-uoa@hippocratio.gr (G.Z.); albanopoulos_Kostis@yahoo.gr (K.A.); 2 School of Health and Caring Sciences, University of West Attica, 12243 Athens, Greece; 3 5th Propaedeutic Surgical Department, Medical School, National and Kapodistrian University of Athens, Eugenideio General Hospital of Athens, 11528 Athens, Greece; evmenenakos@hotmail.com; 4 1st Propaedeutic Surgical Department, Medical School, National and Kapodistrian University of Athens, Laiko General Hospital of Athens, 11527 Athens, Greece; Koszogra92@hotmail.com

**Keywords:** obesity, bariatric surgery, pain dimensions, postoperative pain, preoperative anxiety, preoperative depression

## Abstract

Uncontrolled postoperative pain and prolonged immobilization after bariatric surgery have been associated with increased postoperative complications and prolonged hospitalization. The aim of our study was to evaluate the postoperative pain that follows bariatric surgery and identify any psychological factors that may affect the early postoperative perception of pain. The study included 100 patients with obesity (women, *n* = 61; age 37.4 ± 9.9 years, mean ± standard deviation; Body Mass Index (BMI) 47.6 ± 6.5 kg/m^2^) who underwent bariatric surgery. Preoperative anxiety and depression were evaluated by the Hospital Anxiety and Depression Scale (HADS), and the quantitative and qualitative dimension of early postoperative pain were evaluated by the McGill Pain Questionnaire Short Form (MPQ-SF). Furthermore, the postoperative analgesia protocol was recorded for each patient. Pain declined gradually during the first 24 h postoperative. Although preoperative anxiety had no correlation with the overall pain of postoperative Day 0, patients with a higher level of preoperative anxiety had significantly more intense and more unpleasant pain at 1 h post operation. In addition, depression influences both the intensity and unpleasantness of pain at different time points (1 h, 4 h and 24 h postoperative). Preoperative pain correlated with educational level, but not with age, BMI, gender, marital status, smoking and surgery type. In conclusion, preoperative anxiety and depression influence the early postoperative pain after bariatric surgery, and their preoperative identification is of major importance to enhance the implementation of fast-track postoperative protocols to prevent complications and prolonged hospitalization.

## 1. Introduction

The prevalence of severe obesity, i.e., body mass index (BMI) higher than 35 kg/m^2^, is increasing rapidly in the developing world [1] and has become a global problem [2]. Obesity relates to an increased prevalence of comorbidities [3,4], including type 2 diabetes, cardiovascular diseases, respiratory diseases, back and lower extremity weight-bearing degenerative problems, several forms of cancer, and mental health problems leading to increased mortality [2,4,5]. When conservative methods of obesity treatment fail, bariatric surgery remains the only effective method of weight loss [6,7,8]. Bariatric surgery is gaining more and more popularity since it offers efficient weight loss with proven long-term effects, contributing to improved physical and mental quality of life [9]. The obese population [2,3,10] and the number of bariatric surgeries performed are increasing worldwide [11,12]. Technological advances and progress in surgical techniques have resulted in the adoption of new surgical procedures, classified as restrictive and/or malabsorptive based on the presumed weight loss mechanism [10].

Currently, the most common bariatric procedures worldwide are the laparoscopic Roux-en-Y gastric bypass (LRYGB) and the laparoscopic sleeve gastrectomy (LSG) [11,13]. The one anastomosis gastric bypass (OAGB), also known as single anastomosis gastric bypass or mini gastric bypass, is an effective, safe and simple alternative of LRYGB. Moreover, it has a significant reduction of technical complexity, shorter operative time and a potential reduction in morbidity and mortality [14,15]. Although the laparoscopic surgical approach has the advantage of less postoperative pain [16], patients undergoing laparoscopic bariatric surgery still require efficient analgesia to prevent postoperative complications. Effective pain control reduces the risk of postoperative complications such as pneumonia or atelectasis by enhancing deep breathing, deep vein thrombosis and pulmonary embolism, by encouraging early mobilization [17]. Furthermore, the effective control of perioperative pain helps prevent its transition to chronic pain [14]. A restriction concerning the efficient postoperative analgesia of obese patients is that these patients frequently suffer from comorbidities such as obstructive sleep apnea and other forms of sleep-disordered breathing, making the use of systemic opioids at the early postoperative period very challenging [18]. Postoperative pain management strategies for obesity surgery focuses on ensuring adequate pain relief and mitigating the risk of complications in analgesic use.

Pain is a subjective experience that is characterized by its intensity and its quality characteristics. The quality characteristics of pain are described by words that reflect (a) the sensory qualities of pain in terms of temporal, spatial, pressure, thermal and other properties; and (b) the affective qualities of pain in terms of tension, fear and autonomic properties that are part of the pain experience [19]. The sensory and affective dimensions are considered to be separate and differentially modifiable [20]. A variety of factors are associated with postoperative pain such as patients’ demographic and psychological parameters, different types of surgery and surgical characteristics [21,22]. The correlation between psychological parameters and perception of pain is well established [21], and so is the association between morbid obesity and psychopathological conditions [23]. Preoperative depression and anxiety are two identified psychological factors influencing the experience of postoperative pain in many types of surgeries [24,25,26], but little is known about how they could affect the early postoperative pain following bariatric surgery.

Identifying the psychological factors affecting the postoperative perception of pain for this group of patients may lead to more efficient pain protocols, thus enhancing the implementation of fast-track bariatric protocols [27,28,29]. Therefore, the aim of the present study is to evaluate the early postoperative pain of patients undergoing bariatric surgery and investigate the relationship between pain and the preoperative feeling of anxiety and depression.

## 2. Material and Methods

The study was approved by the Institutional Human Subjects Review Board of our hospital. During a 2-year period (2016–2018), 250 patients with obesity underwent bariatric surgery at our Laparoscopic Bariatric Unit. Our inclusion criteria were as follows: age 18–65 years, BMI ≥ 30 kg/m^2^, laparoscopic bariatric procedure of LSG or OAGB, and patients with I and II physical status according to the classification system of the American Society of Anesthesiologists. The exclusion criteria were history of bariatric or other upper abdominal surgery, diagnosis of a psychiatric disorder, diagnosis of chronic pain, intraoperative placement of a drain, simultaneous cholecystectomy, and postoperative complication and reoperation. In total, 100 patients (women, *n* = 61; age 37.4 ± 9.9 years, mean ± standard deviation; BMI, 47.6 ± 6.5 kg/m^2^) were eligible to participate in our study. The sample size (*n* = 100) of the study was determined using the statistical software G*Power 3.1.9.6 for macOS (https://www.psychologie.hhu.de/arbeitsgruppen/allgemeine-psychologie-und-arbeitspsychologie/gpower.html, Heinrich Heine University Düsseldorf, Düsseldorf, Germany) considering tail one, effect size 0.3, error probability alpha 0.05 and power (1-error probability beta) 0.93 for correlational analysis.

Prior to the experimental procedures, the patients were informed in writing and orally about the surgical procedure and the survey protocol. After signed written consent was obtained, all patients were asked to complete a questionnaire about their medical history, socioeconomic status and the Hospital Anxiety and Depression Scale (HADS). In the intraoperative period, the researcher provided a questionnaire about the surgery and analgesia protocol. All patients were asked to complete the Greek version of the McGill Pain Questionnaire Short Form (MPQ-SF) [30,31] during the postoperative Day 0 (POD #0) at 1, 4, 8, 12 and 24 h after the completion of the operation.

The research protocol predetermined the intraoperative and postoperative analgesia consisting of a bolus intravenous infusion of 1gr paracetamol plus 2 mcg/kg fentanyl 30 min before the animation. Postoperatively, the protocol consisted of an intravenous infusion (IV) of 1gr paracetamol every 6 h, in addition to a fentanyl solution (10 mcg/mL) infusion through a patient-controlled analgesia (PCA) pump, with a stable rate of 2.5 mL/h. Furthermore, the PCA pump allowed patients to self-administer small IV doses of fentanyl (2 mL) with a lockout interval of 10 min. The self-administrated fentanyl volume during the first 24 h was recorded for each patient.

### 2.1. Assessment of Preoperative Psychological Status

HADS is a tool to measure the feeling of anxiety (HADS-A) and depression (HADS-D) [32]. It is used to identify patients in the general hospital who require more systematic psychiatric assessment and care. This tool consists of seven questions that evaluate anxiety and seven questions that evaluate depression. Respondents can answer every question on a four-stage Likert scale scoring from 0 to 3. The scores attributed to the questions are summed up separately for the questions assessing depression and those evaluating anxiety, leading to two scores that range between 0 and 21. High score values indicate high levels of anxiety or depression. Scores between 0–7 points indicate normal levels, scores between 8–10 points indicate border-line abnormal levels and scores between 11–21 points indicate abnormal levels.

### 2.2. Evaluation of Postoperative Pain

Postoperative pain was evaluated by using the validated Greek version of MPQ-SF [30,31]. The questionnaire consists of 15 descriptive adjectives for the pain sensation, 11 sensory and 4 affective, each one assessed by four levels of intensity (none, mild, moderate, severe). Each level scores different points (none = 0, mild = 1, moderate = 2 and severe = 3 points). A sensory and an affective score are calculated by adding the points attributed to the selected adjectives. In addition, a total score (SFMPQ-total) obtained by adding the sensory and the affective score is also calculated. Furthermore, the questionnaire includes a Numeric Rating Scale (NRS), which is a visual horizontal 10 cm analogue scale (VAS) to describe the intensity of pain (0 = no pain, 10 = pain as bad as it could possibly be). A 6-point verbal rating scale pain (Present Pain Index—PPI) is also a component of the MPQ-SF that uses adjectives to describe the feeling of pain at the time of completion of the questionnaire (no pain = 0, mild = 1, discomforting = 2, distressing = 3, horrible = 4 and excruciating = 5). In addition to the use of MPQ-SF, postoperative pain was evaluated by recording the self-administration of small IV doses of fentanyl (2 mL) through the PCA pump during the first 24 h.

### 2.3. Statistical Analysis

Parametric (mean ± SD) and non-parametric statistics (median and interquartile range) described variables. Despite the use of variables described by ordinal scales, we presented data with both parametric and non-parametric statistics to have comparable data with the literature [29,30]. The Spearman correlation coefficient (r) was used to explore the association between two quantitative variables. The correlation is considered low when rho ranges from 0.1 to 0.3, moderate when rho ranges from 0.31 to 0.5 and high when rho is greater than 0.5. Linear regression analysis was used to find independent factors related to the pain scales from which dependence factors (b) and their standard errors (standard errors = SE) were derived. Age, gender, BMI, marital status, smoking, educational level and type of surgery were considered as potential prognostic factors of postoperative pain. The validity of the models, i.e., normality of the residuals, homoscedasticity and multicollinearity, were checked. Statistical significance was set at *p* < 0.05. Analyses were conducted using SPSS statistical software (version 22.0, IBM, Armonk, NY, USA).

## 3. Results

### 3.1. Sample Characteristics

The operations performed were LSG on 58 patients (58%) and laparoscopic OAGB on 42 patients (42%). Table 1 outlines patients’ characteristics and surgical procedures.

### 3.2. Preoperative Psychological Characteristics

The preoperative feeling of anxiety and depression was evaluated using the HADS. With respect to the preoperative level of anxiety, 76% of patients were assessed as having a normal level, 15% as having a border-line level and 9% as having an abnormal level. As for the preoperative depression level, 89% were assessed as having a normal level, 7% as having a border-line level and 4% as abnormal. The mean values of the sample’s preoperative anxiety and depression levels were both normal (0–7 points). The mean preoperative anxiety score was at 4.24 (out of 21), and the mean preoperative depression score at 5.78 (out of 21) (Table 2).

### 3.3. Early Postoperative Pain

At POD #0, the NRS was 4.61 (out of 10), the PPI was 1.79 (out of 5), the sensory score was 6.32 (out of 33), the affective score was 2.81 (out of 12) and the SFMPQ total was 9.16 (out of 45) (Table 3). Although the pain measurements at different time points showed a gradual decrease of pain during the first 24 postoperative hours, distressing (PPI = 3) and severe pain (NRS ≥ 7) was present in the 1st postoperative hour that remained moderate (4 ≤ NRS ≤ 6) until the 8th postoperative hour.

The need for extra bolus infusions of the analgesic drug through the Patient Controlled Analgesia (PCA) pump during the POD #0 was recorded; patients’ attempts were 19.0 ± 14.7, while the amount of analgesic drug delivered from the attempts was 0.4 ± 0.3 mg. Table 4 shows Spearman correlation between the average pain scores for each pain scale NRS, PPI, Sensory, Affective and SFMPQ total with data from PCA such as patient attempts, bolus provided by attempts, bolus provided by continues rate and the total bolus. The amount provided by patients’ attempts was found to be significantly positively correlated with the average intensity score NRS (*p* = 0.042) and the average score on the Sensory (*p* = 0.048), affective (*p* = 0.049) and total emotional score SFMPQ (*p* = 0.043), with patients in more pain seeking extra analgesic drugs by using the PCA pump.

### 3.4. Early Postoperative Pain and Sociodemographic Characteristics/Type of Surgery

The multiple linear regression showed that age, gender, BMI, marital status, smoking and type of surgery did not have any association (positive or negative) with pain ratings for the first 24 postoperative hours. The educational level had a positive association (*p* ≤ 0.05) with all pain parameters’ ratings (NRS, PPI, sensory score, affective score and SFMPQ—total score). Patients with a higher educational level experienced more severe pain both in terms of quality and intensity during the first 24 hours. Furthermore, the multiple linear regression analysis between the bolus infusions of the analgesic drug through the PCA attempts showed no association with all the above characteristics (Table 5).

### 3.5. Early Postoperative Pain and Preoperative Psychological Status

The investigation of the influence of preoperative anxiety or depression on the experience of pain in every predetermined time point revealed an association between psychological factors and pain at different postoperative time points during POD #0. An important positive correlation was observed between the preoperative feeling of anxiety and the pain of the 1st postoperative hour, with most of the questionnaire’s parameters being affected. Patients reporting a higher level of preoperative anxiety experienced severe pain at the 1st preoperative hour in terms of both pain intensity and unpleasantness. In contrast, preoperative feelings of depression seem to influence only the perception of the quality of postoperative pain at the 1st hour (affective score) but extract an important influence on severity and unpleasantness of pain (NRS, PPI, affective score) at the 4th postoperative hour. Furthermore, patients with higher levels of preoperative depression report less severe unpleasantness (affective and SF-MPQ score) at the 24th postoperative pain measurement (Table 6).

## 4. Discussion

Many studies have demonstrated a higher prevalence of psychological disorders among obese patients who are candidates for bariatric surgery [33,34,35]. In our hospital, the preoperative assessment of patients planning to undergo bariatric surgery includes a professional psychiatric evaluation, and for patients with proven pathology, the operation is postponed. A reevaluation is performed after psychiatric treatment, and if the patient is proven to be psychologically stabilized, the operation is performed. This strategy could explain why the preoperative assessment of the feelings of anxiety and depression using HADS classifies most of our patients in the normal range. Nonetheless, 24% and 11% of our patients were assessed as having border-line or abnormal anxiety or depression levels, respectively.

It is well known that psychological factors play a role in the perception of postoperative pain [21,25,36]. This is the first study demonstrating the influence of preoperative anxiety and depression on postoperative pain at different time points of POD #0 after a bariatric surgery. Furthermore, the assessment of pain using a well-structured questionnaire (MPQ-SF) that evaluates both the quantitative and qualitative dimensions of pain permits a detailed description of the perception of postoperative pain.

In this study, pain control during the first eight postoperative hours was insufficient, with patients experiencing severe and distressing pain at the 1st postoperative hour decreasing to moderate pain 4 h after the surgery and persisting until the 8th postoperative hour. Furthermore, anxiety seemed to play a role on pain perception at the 1st postoperative hour, with patients with higher levels of preoperative anxiety experiencing more severe pain in terms of intensity and quality. This finding confirmed the results of previous studies showing that anxiety is a significant positive predictor of severe pain shortly after the operation [37,38]. Moreover, this study extended this knowledge by being the first to evaluate the impact of the preoperative level of anxiety on the qualitative dimension of pain after bariatric surgery. Depression, on the other hand, had a statistically significant positive correlation only with the affective dimension of pain at the 1st postoperative hour. However, depression influenced both quantity and quality characteristics of pain at the 4th postoperative hour, with patients with a higher preoperative level of depression experiencing an intense and unpleasant pain. This finding is also in line with a previous study describing the positive correlation of depression with the intensity of postoperative pain after a bariatric surgery [21]. Our study is the first to show a positive correlation with the intensity of quality characteristics of pain at different time points during POD #0.

An unexpected finding of our study was the impact of preoperative depression on the affective dimension of pain at the 24th postoperative hour measurement. Patients with higher levels of preoperative depression experienced less unpleasantness (affective component) of pain 24 h after the surgery. Moreover, it also influenced the total perception of pain in terms of pain sensation at this time point (SFMPQ—total score). Studies evaluating the experimental pain in depressive patients have reported equivocal results, with some describing increased or decreased pain thresholds [39] depending on the underlying mode of pain application [40] or type of surgery [41,42] and only one examining the affective dimension of experimental pain [43]. At this time point (24th postoperative hour), pain is mild, with the score of the affective component being 1.2 (out of 12) and the score of the SFMPQ-total being 4.1 (out of 45). We can assume that this finding has no clinical significance.

Another interesting finding is that patients’ educational level is an independent factor influencing the perception of quantitative and qualitative characteristics of pain, with patients with higher educational levels reporting more severe pain during the POD #0 after a bariatric surgery. This positive correlation was significant with all the MPQ-SF parameters. On the other hand, age, gender, BMI, marital status, smoking and type of surgery had no influence on postoperative pain. Our findings are supported by the results of other studies about bariatric surgery, which reported that gender, age, surgical technique and smoking are not related to postoperative pain [44,45,46]. However, these results are in contrast with the positive correlations observed in another investigation between age and postoperative pain, at the first 24 h after Laparoscopic gastric by-pass surgery [46]. In terms of educational status, there is no study in bariatric surgery (except in general surgery) demonstrating the impact of educational status on the perception of pain [47]. An informative discussion with patients before surgery may have helped patients with a high or low educational level to have lower pain scores.

## 5. Conclusions

In conclusion, preoperative anxiety and depression and a high educational level are factors influencing the qualitative and quantitative dimension of early postoperative pain during the first postoperative day (POD #0). The preoperative identification of these factors may be useful for designing personalized and time-dependent postoperative analgesic protocols to improve clinical outcomes. Furthermore, regular and systematic pain assessment during different time points at the POD #0 after bariatric surgery is essential for the efficient control of early postoperative pain. The efficient control of early postoperative pain enhances early mobilization and promotes the implementation of fast-track bariatric protocols. This strategy is of major importance due to the susceptibility of bariatric patients to postoperative complications.

## Figures and Tables

**Table 1:** Information about the surgical procedure and descriptions of general and demographic features of the study sample.

Variable		*n* (%)
Gender, *n* (%)	Female	61 (61%)
	Male	39 (39%)
Educational level, *n* (%)	Primary	5 (5%)
	Secondary	53 (53%)
	Two-year degree	18 (18%)
	University	23 (23%)
	Postgraduate university education	1 (1%)
Smoking, *n* (%)	No	52 (52%)
	Yes	35 (35%)
	In the past	13 (13%)
Operation, *n* (%)	LSG	58 (58%)
	OAGB	42 (42%)

BMI, body mass index; LSG, laparoscopic sleeve gastrectomy; OAGB, one anastomosis gastric by-pass.

**Table 2:** Sample’s level of preoperative anxiety and depression.

Preoperative Level	Normal (%)	Border-Line (%)	Abnormal (%)	Mean ± SD	Median (IQR)
Anxiety	76	15	9	4.24 ± 3.09	4 (2–6)
Depression	89	7	4	5.78 ± 3.55	5 (3–7)

SD, standard deviation; IQR = 25th–75th interquartile range.

**Table 3:** McGill Pain Questionnaire Short Form (MPQ-SF) parameters for every predetermined time point and during the first 24 postoperative hours.

	1 h	4 h	8 h	12 h	24 h	Overall Day 0
	Mean ± SD (Median, IQR)	Mean ± SD (Median, IQR)	Mean ± SD (Median, IQR)	Mean ± SD (Median, IQR)	Mean ± SD (Median, IQR)	Mean ± SD (Median, IQR)
**NRS**	7.4 ± 2.1 (8, 6–9)	5.4 ± 2.5 (5, 3–7)	4.3 ± 2.5 (4, 2–6)	3.6 ± 2.4 (3, 1–5)	2.3 ± 2.1 (2, 1–4)	4.61 ± 1.71 (4.8, 3.2–6.0)
**PPI**	3.00 ± 1.27 (3, 2–4)	2.01 ± 0.98 (2, 1–2)	1.58 ± 0.96 (1, 1–2)	1.31 ± 0.79 (1, 1–2)	1.02 ± 0.76 (1, 0.5–1.5)	1.79 ± 0.62 (1.8, 1.4–2.2)
**Sensory**	11.9 ± 6.2 (11, 7–16)	8.1 ± 5.5 (7, 4–11)	4.8 ± 4.8 (4, 2–6)	3.4 ± 3.7 (2, 1.3–4)	3.0 ± 3.4 (2, 0–4)	6.32 ± 3.67 (5.4, 3.8–8.0)
**Affective**	5.4 ± 2.9 (5, 3–8)	3.4 ± 2.5 (3, 2–4)	2.3 ± 2.2 (2, 1–3)	1.6 ± 1.6 (1, 0–2)	1.2 ± 1.6 (1, 0–2)	2.81 ± 1.53 (2.4, 1.8–3.8)
**SFMPQ Total**	17.3 ± 8.5 (16, 10.5–25)	11.5 ± 7.2 (10, 7–15)	7.1 ± 6.4 (6, 3–9)	5.1 ± 5.0 (4, 2–6)	4.1 ± 4.7 (3.5, 1–5)	9.16 ± 4.88 (7.8, 5.6–11.6)

SD, standard deviation; IQR = 25th–5th interquartile range; NRS, Numeric Rating Scale; PPI, Present Pain Index; SFMPQ—total score, Short Form McGill Pain Questionnaire—total score; Day 0, First 24 postoperative hours.

**Table 4:** Correlations between PCA pump amounts and average pain scores for each pain scale during the first 24 postoperative hours.

		NRS	PPI	Sensory	Affective	SFMPQ Total
**Patient attempts**	r	0.20	0.15	0.19	0.19	0.19
	*p*	0.062	0.152	0.075	0.073	0.066
**Patient bolus given** **from attempts (mg)**	r	0.21	0.17	0.21	0.21	0.21
	*p*	0.042	0.117	0.048	0.049	0.043
**Patient bolus given** **from continuous rate (mg)**	r	−0.01	0.10	0.06	−0.03	0.04
	*p*	0.962	0.330	0.599	0.780	0.725
**Total bolus given(mg)**	r	0.10	0.16	0.15	0.12	0.15
	*p*	0.347	0.132	0.157	0.240	0.163

PCA: Patient Controlled Analgesia, NRS: Numerical Rating Scale, PPI: Present Pain Index, SFMPQ—total score: Short Form McGill Pain Questionnaire—total score.

**Table 5:** Correlations between all pain scales with educational level of participants.

Pain Scale	Educational Level	b ^+^	SE ^++^	*p*
NRS	Primary			
	Secondary	0.142	0.046	0.003
PPI	Primary			
	Secondary	0.125	0.045	0.007
Sensory	Primary			
	Secondary	0.117	0.057	0.043
Affective	Primary			
	Secondary	0.125	0.065	0.050
SFMPQ total	Primary			
	Secondary	0.110	0.055	0.047

^+^ Dependency factor, ^++^ standard factor error. NRS: Numeric Rating Scale, PPI: Present Pain Index, SFMPQ—total score: Short Form McGill Pain Questionnaire—total score.

**Table 6:** Spearman’s correlation coefficients of the preoperative anxiety and depression scores with the MPQ-SF parameters for every predetermined time point.

	Postoperative Hour	NRS		PPI		Sensory Score		Affective Score		SFMPQ Total Score	
		r	*p*	r	*p*	r	*p*	r	*p*	r	*p*
**Preoperative Anxiety**	1st hour	0.22	0.030	0,.04	0.676	0.28	0.005	0.29	0.004	0.31	0.002
	4th hour	0.04	0.731	−0.02	0.850	0.12	0.257	0.07	0.514	0.13	0.215
	8th hour	0.04	0.675	0.00	0.987	0.06	0.550	−0.06	0.540	0.02	0.844
	12th hour	−0.03	0.744	−0.07	0.508	−0.02	0.868	−0.05	0.653	−0.02	0.812
	24th hour	−0.07	0.516	−0.08	0.424	−0.02	0.857	−0.10	0.340	−0.07	0.494
**Preoperative Depression**	1st hour	0.14	0.158	0.04	0.662	0.14	0.181	0.22	0.034	0.17	0.096
	4th hour	0.21	0.038	0.20	0.046	0.14	0.185	0.22	0.029	0.16	0.110
	8th hour	0.10	0.303	0.01	0.906	0.05	0.625	0.10	0.319	0.06	0.531
	12th hour	−0.02	0.871	−0.03	0.755	0.04	0.710	0.04	0.690	0.06	0.569
	24th hour	0.05	0.644	0.00	0.994	−0.09	0.395	−0.28	0.004	−0.23	0.038

NRS: Numeric Rating Scale, PPI: Present Pain Index, SFMPQ—total score: Short Form McGill Pain Questionnaire—total score.

## Data Availability

All data are available by the corresponding author (S.G.) upon reasonable request.

## References

[1] Wang M., Xu S., Liu W., Zhang C., Zhang X., Wang L., Liu J., Zhu Z., Hu J., Luo X. (2020). Prevalence and changes of BMI categories in China and related chronic diseases: Cross-sectional National Health Service Surveys (NHSSs) from 2013 to 2018. EClinicalMedicine.

[2] Di Angelantonio E., Bhupathiraju S.N., Wormser D., Gao P., Kaptoge S., de Gonzalez A.B., Cairns B.J., Huxley R., Jackson C.L., Joshy G. (2016). Body-mass index and all-cause mortality: Individual-participant-data meta-analysis of 239 prospective studies in four continents. Lancet.

[3] Residori L., García-Lorda P., Flancbaum L., Pi-Sunyer F.X., Laferrére B. (2003). Prevalence of co-morbidities in obese patients before bariatric surgery: Effect of race. Obes. Surg..

[4] Buchwald H., Avidor Y., Braunwald E., Jensen M.D., Pories W., Fahrbach K., Schoelles K. (2004). Bariatric surgery: A systematic review and meta-analysis. J. Am. Med. Assoc..

[5] Pantalone K.M., Hobbs T.M., Chagin K.M., Kong S.X., Wells B.J., Kattan M.W., Bouchard J., Sakurada B., Milinovich A., Weng W. (2017). Prevalence and recognition of obesity and its associated comorbidities: Cross-sectional analysis of electronic health record data from a large US integrated health system. BMJ Open.

[6] Noria S.F., Grantcharov T. (2013). Biological effects of bariatric surgery on obesity-related comorbidities. Can. J. Surg..

[7] Picot J., Jones J., Colquitt J.L., Gospodarevskaya E., Loveman E., Baxter L., Clegg A.J. (2009). The clinical effectiveness and cost-effectiveness of bariatric (weight loss) surgery for obesity: A systematic review and economic evaluation. Health Technol. Assess..

[8] Douglas I.J., Bhaskaran K., Batterham R.L., Smeeth L. (2015). Bariatric Surgery in the United Kingdom: A Cohort Study of Weight Loss and Clinical Outcomes in Routine Clinical Care. PLoS Med..

[9] Raaijmakers L.C.H., Pouwels S., Thomassen S.E.M., Nienhuijs S.W. (2017). Quality of life and bariatric surgery: A systematic review of short- and long-term results and comparison with community norms. Eur. J. Clin. Nutr..

[10] NCD Risk Factor Collaboration (NCD-RisC) (2016). Trends in adult body-mass index in 200 countries from 1975 to 2014: A pooled analysis of 1698 population-based measurement studies with 19.2 million participants. Lancet.

[11] Angrisani L., Santonicola A., Iovino P., Formisano G., Buchwald H., Scopinaro N. (2015). Bariatric Surgery Worldwide 2013. Obes. Surg..

[12] Parker M., Loewen M., Sullivan T., Yatco E., Cerabona T., Savino J.A., Kaul A. (2007). Predictors of outcome after obesity surgery in New York state from 1991 to 2003. Surg. Endosc..

[13] Kozlowski T., Kozakiewicz K., Dadan J., Mysliwiec P. (2016). Innovative solutions in bariatric surgery. Gland Surg..

[14] Chaim E.A., Ramos A.C., Cazzo E. (2017). Mini-Gastric Bypass: Description of the Technique and Preliminary Results. Arq. Bras. Cir. Dig..

[15] Carbajo M.A., Luque-de-León E., Jiménez J.M., Ortiz-de-Solórzano J., Pérez-Miranda M., Castro-Alija M.J. (2017). Laparoscopic One-Anastomosis Gastric Bypass: Technique, Results, and Long-Term Follow-Up in 1200 Patients. Obes. Surg..

[16] El Shobary H., Christou N., Backman S.B., Gvocdic B., Schricker T. (2006). Effect of laparoscopic versus open gastric bypass surgery on postoperative pain and bowel function. Obes. Surg..

[17] Gan T.J. (2017). Poorly controlled postoperative pain: Prevalence, consequences, and prevention. J. Pain Res..

[18] Adams J.P., Murphy P.G. (2000). Obesity in anaesthesia and intensive care. Br. J. Anaesth..

[19] Melzack R., Katz J. (2011). The McGill pain questionnaire: Development, psychometric properties, and usefulness of the long form, short form, and short form-2. Handbook of Pain Assessment.

[20] Auvray M., Myin E., Spence C. (2010). The sensory-discriminative and affective-motivational aspects of pain. Neurosci. Biobehav. Rev..

[21] Ip H.Y.V., Abrishami A., Peng P.W.H., Wong J., Chung F. (2009). Predictors of postoperative pain and analgesic consumption: A qualitative systematic review. Anesthesiology.

[22] Yang M.M., Hartley R.L., Leung A.A., Ronksley P.E., Jetté N., Casha S., Riva-Cambrin J. (2019). Preoperative predictors of poor acute postoperative pain control: A systematic review and meta-analysis. BMJ Open.

[23] Malik S., Mitchell J.E., Engel S., Crosby R., Wonderlich S. (2014). Psychopathology in bariatric surgery candidates: A review of studies using structured diagnostic interviews. Compr. Psychiatry.

[24] Kavaki O., Altuntas E., Muderris S. (2012). Effects of the preoperative anxiety and depression on the postoperative pain in ear, nose, and throat surgery. Indian J. Otol..

[25] Sobol-Kwapinska M., Bąbel P., Plotek W., Stelcer B. (2016). Psychological correlates of acute postsurgical pain: A systematic review and meta-analysis. Eur. J. Pain..

[26] Periañez C.A.H., Diaz M.A.C., Bonisson P.L.V., Simino G.P.R., Barbosa M.H., de Mattia A.L. (2020). Relationship of anxiety and preoperative depression with post-operative pain. Texto Contexto Enferm..

[27] Elliott J.A., Patel V.M., Kirresh A., Ashrafian H., Le Roux C.W., Olbers T., Athanasiou T., Zacharakis E. (2013). Fast-track laparoscopic bariatric surgery: A systematic review. Updates Surg..

[28] Bamgbade O.A., Oluwole O., Khaw R.R. (2017). Perioperative Analgesia for Fast-Track Laparoscopic Bariatric Surgery. Obes. Surg..

[29] Nijland L.M.G., de Castro S.M.M., van Veen R.N. (2020). Risk Factors Associated with Prolonged Hospital Stay and Readmission in Patients After Primary Bariatric Surgery. Obes. Surg..

[30] Georgoudis G., Watson P.J., Oldham J.A. (2000). The development and validation of a Greek version of the short-form McGill Pain Questionnaire. Eur. J. Pain.

[31] Georgoudis G., Oldham J.A., Watson P.J. (2001). Reliability and sensitivity measures of the greek version of the short form of the McGill Pain Questionnaire. Eur. J. Pain.

[32] Zigmond A.S., Snaith R.P. (1983). The Hospital Anxiety and Depression Scale. Acta Psychiatr. Scand..

[33] Van Hout G.C.M., Van Oudheusden I., Van Heck G.L. (2004). Psychological profile of the morbidly obese. Obes. Surg..

[34] Marzocchi R., Moscatiello S., Villanova N., Suppini A., Marchesini G. (2008). Psychological Profile and Quality of Life of Morbid Obese Patients Attending a Cognitive Behavioural Program. Psihol. Teme.

[35] McGarrity L.A., Perry N.S., Derbidge C.M., Trapp S.K., Terrill A.L., Smith T.W., Ibele A.R., MacKenzie J.J. (2019). Associations Between Approach and Avoidance Coping, Psychological Distress, and Disordered Eating Among Candidates for Bariatric Surgery. Obes. Surg..

[36] Hah J.M., Hilmoe H., Schmidt P., McCue R., Trafton J., Clay D., Sharifzadeh Y., Ruchelli G., Boussard T.H., Goodman S. (2020). Preoperative factors associated with remote postoperative pain resolution and opioid cessation in a mixed surgical cohort: Post hoc analysis of a perioperative gabapentin trial. J. Pain Res..

[37] Kalkman C.J., Visser K., Moen J., Bonsel G.J., Grobbee D.E., Moons K.G.M. (2003). Preoperative prediction of severe postoperative pain. Pain.

[38] Kain Z.N., Sevarino F., Alexander G.M., Pincus S., Mayes L.C. (2000). Preoperative anxiety and postoperative pain in women undergoing hysterectomy: A repeated-measures design. J. Psychosom. Res..

[39] Dickens C., McGowan L., Dale S. (2003). Impact of depression on experimental pain perception: A systematic review of the literature with meta-analysis. Psychosom. Med..

[40] Bär K.J., Brehm S., Boettger M.K., Boettger S., Wagner G., Sauer H. (2005). Pain perception in major depression depends on pain modality. Pain.

[41] Caumo W., Schmidt A.P., Schneider C.N., Bergmann J., Iwamoto C.W., Adamatti L.C., Bandeira D., Ferreira M.B.C. (2002). Preoperative predictors of moderate to intense acute postoperative pain in patients undergoing abdominal surgery. Acta Anaesthesiol. Scand..

[42] Pinto P.R., McIntyre T., Araújo-Soares V., Costa P., Almeida A. (2015). Differential Predictors of Acute Post-Surgical Pain Intensity After Abdominal Hysterectomy and Major Joint Arthroplasty. Ann. Behav. Med..

[43] Dworkin R.H., Clark W.C., Lipsitz J.D. (1995). Pain responsivity in major depression and bipolar disorder. Psychiatry Res..

[44] Ferreira A.T., Duarte N.M., Caetano A.M., Albuquerque K.A., Buenos Aires V., Brainer-Lima J.P., Hinrichsen E.A., Santa-Cruz F., Campos J.M. (2019). Postoperative pain following bariatric surgery: Correlation between intensity and clinical-surgical variables. Bariatr. Surg. Pract. Patient Care.

[45] Karlnoski R.A., Puri S., Chen R., Mangar D., Murr M.M., Camporesi E.M. (2013). Reduced postoperative pain and complications after a modified multidisciplinary approach for bariatric surgery. Open Obes. J..

[46] Hartwig M., Allvin R., Bäckström R., Stenberg E. (2017). Factors Associated with Increased Experience of Postoperative Pain after Laparoscopic Gastric Bypass Surgery. Obes. Surg..

[47] Lanitis S., Mimigianni C., Raptis D., Sourtse G., Sgourakis G., Karaliotas C. (2015). The impact of educational status on the postoperative perception of pain. Korean J. Pain.

